# Involvement of Tetraspanin C189 in Cell-to-Cell Spreading of the Dengue Virus in C6/36 Cells

**DOI:** 10.1371/journal.pntd.0003885

**Published:** 2015-07-01

**Authors:** Chao-Fu Yang, Cheng-Hsun Tu, Yin-Ping Lo, Chih-Chieh Cheng, Wei-June Chen

**Affiliations:** 1 Graduate Institute of Biomedical Science, College of Medicine, Chang Gung University, Kwei-San, Tao-Yuan, Taiwan; 2 Department of Public Health and Parasitology, College of Medicine, Chang Gung University, Kwei-San, Tao-Yuan, Taiwan; Colorado State University, UNITED STATES

## Abstract

Dengue virus (DENV) is naturally transmitted by mosquitoes to humans, infecting cells of both hosts. Unlike in mammalian cells, DENV usually does not cause extremely deleterious effects on cells of mosquitoes. Despite this, clustered progeny virions were found to form infection foci in a high density cell culture. It is thus interesting to know how the virus spreads among cells in tissues such as the midgut within live mosquitoes. This report demonstrates that cell-to-cell spread is one way for DENV to infect neighboring cells without depending on the “release and entry” mode. In the meantime, a membrane-bound vacuole incorporating tetraspanin C189 was formed in response to DENV infection in the C6/36 cell and was subsequently transported along with the contained virus from one cell to another. Knockdown of C189 in DENV-infected C6/36 cells is shown herein to reduce cell-to-cell transmission of the virus, which may be recovered by co-transfection with a C189-expressing vector in DENV-infected C6/36 cells. Moreover, cell-to-cell transmission usually occurred at the site where the donor cell directly contacts the recipient cell. It suggested that C189 is crucially involved in the intercellular spread of progeny viral particles between mosquito cells. This novel finding presumably accounts for the rapid and efficient infection of DENV after its initial replication within tissues of the mosquito.

## Author Summary

Dengue fever is one of the most important mosquito-borne viral infectious diseases in the world. Its etiological agent is naturally transmitted via blood feeding by *Aedes* mosquitoes. An ingested virus can replicate and be disseminated within and between tissues in mosquitoes. In this study, we found that infection of DENV in C6/36 mosquito cells can stimulate the up-regulation of tetraspanin C189, which usually co-localizes but does not directly interact to form C189-containing membrane-bound vacuoles (C189-VCs). Our results also showed that the virus can be delivered to a neighboring cell along with C189-VCs, frequently through cell contact with filopodia extended by the donor cell that touch the recipient cell. Knockdown of C189 can reduce the efficiency of virus delivery, indicating its crucial role in cell-to-cell transmission of DENV in C6/36 cells. Cell-to-cell transmission may thus be an alternative route for the efficient intercellular spread of progeny viruses within tissues of the mosquito.

## Introduction

Dengue virus (DENV) consists of four serotypes that manifest similar symptoms, ranging from a mild febrile illness to a life-threatening dengue hemorrhagic fever [1]. Taxonomically, DENV is one of some 70 members of the family Flaviviridae and is transmitted between humans by *Aedes* mosquitoes [2], particularly *Aedes aegypti* [3]. Dengue fever (DF) and dengue hemorrhagic fever (DHF)/dengue shock syndrome (DSS) have become increasingly important public health problems in over 100 countries in tropical and subtropical regions [4]. It is estimated that 2.5–3 billion people are risk of dengue infection in the world [5]. As DENV is naturally transmitted to humans by mosquitoes, indicating the virus can also infect and replicate in the mosquito cell during its journey from the midgut to salivary glands [6]. In humans bitten by an infected mosquito, DENV inoculated with mosquito saliva initially infects Langerhan cells and keratocytes residing in the epidermis where it begins to replicate [7]. Subsequently, the virus can infect other organs including circulatory macrophages/monocytes, lymphoid tissues, liver, spleen, kidneys, and lungs [8], as well as the brain in a few cases [9]. DENV has also been detected in megakaryocyte progenitors and circulating platelets [10], suggesting that thrombocytopenia in dengue patients is closely associated with DENV infection [11, 12]. Such host cells are usually infected by DENV through receptor(s)-mediated endocytosis [13] and mostly end up undergoing apoptosis in response to dengue virus infection [14]. A huge number of viral particles from infected cells burst into the blood stream or a culture to become the source of infection for other cells.

Since mosquito cells can be protected from dengue virus infection by way of an induced antioxidant defense as well as anti-apoptotic effects [15, 16], infected cells usually remain intact even when abundant progeny viral particles have been produced within the cell [17]. In mosquito cell cultures, progeny viral particles are also released from infected cells into the medium as in mammalian cells [17]. Like other insects, the mosquito possesses an intestine composed of a monolayer of epithelial cells resting on an extracellular basal lamina that is morphologically divided into three parts; *i*.*e*., the foregut, midgut, and hindgut [18]. Normally, DENV must infect epithelial cells in the midgut, which is the first site of stay for engorged blood meals [19]. DENV contained within the blood meal initially infects a few epithelial cells, followed by the formation of infection foci involving multiple cells. Infection foci gradually expand and the entire organ may become infected within several days post-infection (pi) [20]. This suggests that the virus spreads laterally from the initial site of infection to neighboring cells rather than through a process of “release-and-entry,” leading to an increased number of infected cells that successfully establish the infection in the mosquito [21]. In turn, there might be a unique mechanism other than the process of virus release and entry such as occurs in the mammalian circulatory system or cell cultures for the intercellular spread of the virus in the mosquito vector [22].

Cell-to-cell spread has been reported as a route of rapid dissemination implemented by a variety of animal viruses [23]. Herpesviruses (HSV) are the typical example of spread by direct cell-to-cell transmission [24], which can promote immunity evasion deriving from the effect of a neutralizing antibody that blocks reinfection of other cells by an extracellular virus [23]. Hepatitis C virus (HCV) establishes infection via cell-to-cell transmission in the liver [25], particularly in patients with chronic infection [26]. In the case of human immunodeficiency virus (HIV), infection of CD4^+^ memory T cells are initially driven by cell-free virions. However, direct transfer of infection in the cell-to-cell mode is believed to occur more efficiently and rapidly [27]. Moreover, tetraspanin CD81 is involved in cell-to-cell transmission by such viruses as HIV [26, 28], raising the question of whether DENV can efficiently spread or disseminate via an intercellular mode in tissues, such as the midgut, of a mosquito host.

As mentioned above, most mosquito cells infected by DENV do not exhibit obvious damage but can cause persistent infection [17]. Thus, release-and-entry of the virus in mosquitoes may not be the primary way between cells within a tissue. In contrast, spread by way of cell-to-cell transmission seems to be more logical for extensive and efficient viral infection. In this study, we designed different cell culture systems to reveal how DENV can spread between C6/36 cells in a cell-to-cell transmission mode. As a novel tetraspanin C189 was previously identified from DENV-infected C6/36 cells and was shown a close association with a vacuole containing viral proteins [29], this study also aimed to demonstrate the possibility of its involvement in virus spread between mosquito cells.

## Materials and Methods

### Cell culture and virus

The protocol for cell culture used in this study mostly followed the description reported previously. In brief, DENV type 2 (New Guinea C) used in this study was propagated in *Ae*. *albopictus* C6/36 cells that were grown in minimal essential medium (MEM) (Invitrogen, Carlsbad, CA) with non-essential amino acids and 10% fetal bovine serum (FBS) at 28°C in a closed incubator. Titration of the virus was carried out by plaque assay on BHK-21 cells, which were cultured at 37°C in an incubator with a 5% CO_2_ atmosphere [15].

### Methylcellulose overlay assay

C6/36 cells were dispended to culture in 48-well plates overnight; ~70 μl virus suspension (MOI = 1 or less) was then added to each well. After adsorption for 1 h at 28°C, the virus suspension was removed from each well and replaced with fresh medium containing 5% FBS or with 1.1% methylcellulose medium (a mixture of 2.2% methylcellulose and fresh medium). Subsequently, 1 ml of 4% paraformaldehyde was added to each well after incubation for 24 or 48 h and then the medium or methylcellulose was removed and fixed for 30 min. The plate was washed with PBS twice and then treated with 0.1% Triton X-100 at 4°Cfor 2 min to increase cell membrane permeability. The plate was washed again twice with PBS and then 0.2 ml of 1% BSA was added to each well to block at room temperature (RT) for 1 h or at 4°C overnight. An immunofluorescence assay (IFA) was subsequently implemented as described below.

### Extraction of RNA and first-strand cDNA synthesis

Total RNA was isolated from the pellet of 4 x 10^6^ ~ 1 x 10^7^ C6/36 cells using Trizol reagent (Invitrogen). First-strand cDNA was synthesized using total RNA as the template, and a SuperScript First-Strand Synthesis kit (Invitrogen) was used according to the manufacturer instructions.

### Viral RNA detection by reverse transcriptase-polymerase chain reaction (RT-PCR)

DENV-infected donor cells (MOI = 1, 24 hpi) and eGFP-expressing recipient cells were co-cultured together or separated by transwell either with or without treatment with antiserum for 6 h. Recipient cells were subsequently sorted by flow cytometry and viral RNA was analyzed by RT-PCR. The viral RNA expression level was detected with the primer pair located at the 5’-UTR, including DV2-5UTR-F (TGGACCGACAAAGACAGATTCTT) and DV2-5UTR-R (CGYCCYTGCAGCATTCCAA). The internal control gene 18S was detected with primers 18SF (AGGTCCGTGATGCCCTTAGA) and 18SR (TACAATGTGCGCAGCAACG). The C189 expression level was normalized to the 18S expression level.

### Real time RT-PCR for quantitation of C189 expression level

Total RNA extraction followed the procedure in the previous report using Trizol reagent (Invitrogen, Carlsbad, USA) [29]. The gene expression level was quantified by real-time RT-PCR using SYBR Green dye (Applied Biosystem, Carlsbad, USA). The C189 expression level was detected with primers C189F (CTGCATGACCACGACCTATGG) and C189R (AGAGCGGCAACGACGATTT). 18S was used for the internal control as above.

### Endogenous C189 detection by immunofluorescence assay (IFA)

Infected and uninfected C6/36 cells were smeared on a cover glass and washed with phosphate buffered saline (PBS; pH 7.4) three times. Cells were subsequently fixed in 4% paraformaldehyde for 10 min, washed again with PBS, and then blocked with 1% BSA in PBS for 1 h at 37°C. Primary polyclonal antibodies (1:100 in dilution) against C189 LEL (S1 Text) were added onto the cover glass and incubated at 37°C for 1 h. The cover glass was subsequently incubated with secondary antibodies (1:100) conjugated with FITC at 37°C for 1 h after being washed with PBS. The cover glass was finally mounted with a mixture of glycerol and PBS (3:7) and observed under a laser scanning confocal microscope (Zeiss LSM 510, Carl-Zeiss, Jena, Germany). Negative controls were incubated with diluents without primary antibodies; otherwise, samples were subjected to all the procedures described above.

### Construction of the expression vector

The expression vector used in this study was based on insect-cell-expression vector pAC5.1-V5-His A (Invitrogen), from which pAC5.1-eGFP expression vector was previously constructed (Lin et al., 2007). For the expression of C189 tagged with eGFP fusion proteins, the open reading frame of C189 was amplified by the indicated PCR primers and cDNA derived from C6/36 cells, which were subsequently inserted into the pAC5.1-eGFP expression vector in the N-terminal domain of eGFP [29]. To express red Rhodamine fluorescent protein (RFP), the RFP gene was amplified from pTagRFP-C (Evrogen, Moscow, Russia) and inserted into pAC5.1-V5-His A. To express HAeGFP and HAC189, primers HA-F and HA-R were hybridized and ligated with pAC5.1-V5-His A to generate pAC5.1-HA. eGFP and C189 genes were amplified using the indicated primers and C6/36 cDNA, then inserted into pAC5.1-HA to form HAeGFP and HAC189 expression vectors. The primers to amplify the corresponding genes are listed in S1 Table.

### Transfection of the expression vector into C6/36 cells

C6/36 cells were seeded into 6-well plates and grown to 70–80% confluence for transfection. X-tremeGene HP DNA transfection reagent (Roche, Indianapolis, IN, USA) was mixed with vectors (ratio = 3:1 μl/μg, 1 μg plasmid DNA per well in most experiments) in basal medium (MEM, 2% non-essential amino acid, 0.0375% sodium bicarbonate, 0.2% hepes) at RT for 15 min. Cells were incubated with the transfection mixture for 5 h before replacing with complete medium.

### Establishment of a stable knockdown system in C6/36 cells

A stable knockdown system was based on the miRNA system [30]. The target sequence (83–103 bp) was selected by BLOCK-iT RNAi Designer (Invitrogen). The sequence design and predicted pre-miRNA sequence structure of miC189 are shown in Supplementary S1 Table. After hybridization, design DNA was inserted into miRNA expression vector, pcDNATM6.2-GW/EmGFP-miR (Invitrogen). The pAC5.1-V5-His A was combined with pIE1-neo (Novagen, Cambridge MA, USA) to generate insect-cell-stable-expression vector, pAC5.1-neo. Sequentially, the trans-element of miRNA expression vector was amplified and inserted into pAC5.1-neo. A schematic process for construction of stable knockdown systems was shown in S2 Text. To generate those stable knockdown cell clones, miC189 and miN (negative control provided by Invitrogen) expression vectors were transfected into C6/36 cells and selected with G418 (Invitrogen). After sorting by flow cytometry and serial dilution, 5–10 stable knockdown clones were acquired. The different stable clones had been tested and shown similar knockdown effects.

### Recovery of C189 expression in a miC189 stable clone of C6/36 cells

The miC189 stable clone of C6/36 cells were transfected with a mixture (0.5 μg pAC5.1-C189eGFP and 4 μl FuGENE HD), forming miC189/C189 cells. Subsequently, 1.5 × 10^5^ C6/36 cells, as well as stable clones of miN, miC189, and miC189/C189, were dispensed to each well of the 48-well plates and incubated for 24 h. DENV-2 (1.5 × 10^1^ PFUs) was then inoculated with cells in each well at MOI = 0.0001. After incubation at 28°Cfor 1 h, the virus suspension was removed from each well and 1.1% methylcellulose containing 5% FBS medium was added to the wells. Plates were washed with PBS at 72 hpi and then 1 ml 4% paraformaldehyde was added to each well to fix cells for 20 min. Cells were washed again with PBS and then treated with 0.1% Triton X-100 at RT for 2 min in order to increase cell membrane permeability. Cells in plates were blocked with 0.2 ml 3% BSA at RT for 1 h or at 4°C overnight after another wash with PBS before being subjected to an immunofluorescence antibody test and observed under a fluorescent microscope.

### Detection of the intercellular spread of virus in the transwell system

C6/36 cells (2 × 10^5^ cells/well) transfected with pAC5.1-RFP (or-eGFP), used as recipient cells, were seeded onto the 24-well plat. Another batch of C6/36 cells were infected by DENV-2 (MOI = 1) for 24 h serving as donor cells, those cells were then scratched out and transferred to upper layer of the transwell system (pore size is 4 μm) after being washed five times with PBS. In this system, recipient and donor cells were separated but not limited for virus diffusion movement between layers. To scavenge released virus particles, complement-inactivated serum containing neutralizing bodies diluted to 1:200 from human dengue patients was added, incubated at 4°C for 1 h and then 28°C for 18 h (or 6 h for that referred to RT-PCR). Those not treated with neutralizing bodies served as controls. Recipient cells were subsequently washed with PBS three times, fixed with 4% paraformaldehyde, and then reacted with monoclonal anti-DENV2 NS3 antibodies (1:100). This was followed by a reaction with anti-mouse Alexa Fluor 633 nm (1:100) and observed under a laser confocal microscope (Zeiss LSM510). For RNA identification, recipient cells harvested from the plate were subjected to RNA extraction using Trizol reagents under the protocol mentioned above. RT-PCR was thus carried out using primers (D1 and D2) [31] to detect viral RNA while the detection of 18S rRNA was utilized as the internal control as mentioned above.

### Detection of the intercellular spread of virus in the co-culture system

To establish DENV2-infected donor cells, C6/36 cells were infected by the virus at an MOI of 1 for 24 h after transfection with eGFP expression vectors, C189eGFP expression vectors, miRNA-based knockdown vector miN and miRNA-based knockdown vector miC189, respectively. Another batch of C6/36 cells transfected with RFP expression vector was used as recipient cells. Donor cells washed by PBS to remove cell-free virus were co-cultured with recipient cells in the antiserum-containing medium (the ratio of donor cells to recipient cells was 1 to 3). At 18 h after co-culture, cells were conducted with immunofluorescence assay by fixing and staining with anti-NS3 antibody. As miRNA-based knockdown vectors contain the reporter gene EmGFP which is able to be the marker of donor cells. For viral RNA detection, infected cells scratched out from co-culture containing recipient cells soon after treatment with trypsin. Those cells that positively expressed eGFP were sorted out via flow cytometry and then subjected to RNA extraction, and viral RNA detection was carried out by RT-PCR as described above.

### Immunoprecipitation (IP)

An anti-HA immunoprecipitation kit (Sigma, MO, USA) was utilized for this experiment. In brief, C6/36 cells were cultured in a 6-well plate until about 80% of a monolayer was formed. After transfection with pAC5.1-HAC189 (0.5 μg/well), cells were washed with culture medium once and then infected with a virus suspension at MOI = 1. Plates were incubated at 28°C for 1 h with periodical, gentle shaking for adsorption. Subsequently, 3 ml culture medium was added into each well of the plate, which was incubated again at 28°C for 48 h. After the medium was removed from wells and washed with PBS twice, 100 μl CelLytic-M Cell Lysis Reagent (Sigma) was added into each well and incubated at 4°C for 20 min. Cell lysate was transferred to a microtube and kept at -80°C for 1 h and then centrifuged at 12000 *g* for 10 min at 4°C after re-melting. The supernatant was transferred to a spin column in which 20 μl anti-HA-agarose was added and gently shaken at 4°C for 24 h. The spin column was then put in the collection tube. Liquid in the collection tube was discarded after centrifugation at 4°C and 13000 *g* for 30 sec. Then, 700 μl 1X IP buffer was added into the spin column, centrifuged at 4°C and 13000 *g* for 30 sec, and the liquid in the collection tube was discarded. After repeating this step for five times, 700 μl PBS was added and centrifuged again at 4°C and 13000 *g* for 30 sec. The spin column was then transferred to a new microtube. After discarding the liquid in the collection tube, an equal volume of sample buffer was added into the tube, heated at 95°C for 5 min in a dry bath, and then centrifuged at RT and 13000 *g* for 2 min. The collected product was subjected to analysis by Western blot as described below.

### Western blot

Harvested proteins were electrophoretically separated by 12% (w/v) SDS-PAGE in non-reducing conditions and transferred to Immobilon-P Transfer Membrane (Millipore, Darmstadt, Germany). After blocking with 5% milk-TBS-0.1% Tween 20 buffer at RT for 1 h, the membrane was stained with the indicated primary and secondary antibodies at RT for 1 h, as done previously in this lab. After final washing, the membrane was treated with Western Lightning Chemiluminescence Plus Reagent (PerkinElmer, Waltham, MA, USA) and signals were detected by FUJI X-ray film.

### Co-localization of C189/viral proteins detected through sucrose gradient ultracentrifugation

This experiment followed a method previously reported [32]. Briefly, C6/36 cells were cultured in a 6-well plate until an ~80% monolayer was formed. After transfection with pAC5.1-HAC189 or pAC5.1-eGFP (1 μg/well), cells in each well were infected with a virus suspension for 48 h at MOI = 1. Cells were subsequently scratched from each well and then added into the break buffer (10% w/v sucrose, 1 mM EDTA, 10 mM HEPES, 5 mM MgCl_2_, pH 7.4), and then homogenized on ice for at least 100 strokes in a homogenizer. The homogenized cell lysate was centrifuged at 4°C and 800 *g* for 10 min to remove nuclei and cell debris. The supernatant was topped up with a 10~60% sucrose gradient (10~60% w/v sucrose, 1 mM EDTA, 10 mM HEPES, 5 mM MgCl_2_, pH 7.4) in a tube and ultra-centrifuged at 4°C at 14440 *g* for 18 h. After centrifugation, 17 fractions (0.5 ml each) were collected from the top to the bottom. Of which, the first 7 fractions were pooled to one fraction as their similarity in density. As a result, a total of 11 samples were subject for further analysis by Western blot as described above.

### Imaging demonstration of C189-involved cell-to-cell spread in C6/36 cells

C6/36 cells expressing C189eGFP with dengue 2 virus infection (MOI = 1) for 24 h were used as donor cells. These were washed with PBS and then scratched out to co-culture with recipient cells prepared as above with medium contain neutralizing antibodies derived from human serum. Co-cultured cells were adsorbed on a coverslip after incubating at 4°C for 1 h and then transferred to an 28°C incubator for another 6 h. Ultimately, antiDENV-2 E antibody (1:100) and anti-mouse Alexa Fluor 633 nm (1:100) were applied to IFA and observed under a laser scanning confocal microscope (Zeiss LSM510).

### Real-time monitoring for C189-involved intercellular spread of virus

Uninfected C189eGFP-expressing C6/36 cells (donor cells) and RFP-expressing cells (recipient cells) were co-cultured for 30 h and then subjected to real-time observations on C189 transmission between live cells under a multi-photon confocal microscope (Zeiss LSM510 meta) at an interval of 15 sec. Donor and recipient cells were prepared as above. To observe the transmission of DENV/C189 complexes, infected C189eGFP-expressing (donor) and RFP-expressing (recipient) cells were co-cultured at 4°C for 1 h and then transferred to a 28°C incubator for another 6 h. Co-cultured cells were then observed for C189-mediated cell-to-cell transmission under a confocal microscope.

### Electron microscopy

For electron microscopy, C6/36 cells (harvested 6, 12, 18, or 24 h post-infection with DENV-2) seeded on a dish or scraped from a culture dish (centrifugation at 4°C and 3000 rpm for 10 min) were immediately fixed with a mixture of 2% (v/v) glutaraldehyde and 2% paraformaldehyde in 0.1 M cacodylate buffer overnight at 4°C. After post-fixing in 1% (w/v) osmium tetroxide in 0.1 M cacodylate buffer for 2 h at room temperature, cells were washed with 0.2 M cacodylate buffer three times. Cells were again washed with 0.2 M cacodylate buffer three times and then dehydrated through an ascending series of ethanol grades. Cells were finally embedded in Spurr's resin (Electron Microscopy Science, Hatfield, PA, USA) and polymerized at 70°C for 72 h. Trimmed blocks were sectioned with an ultramicrotome (Reichert Ultracut R, Leica, Vienna, Austria). Immunocytochemistry embedding used LR White resin (London Resin Co. Ltd., Basingstoke, Hampshire, England) followed by treatment with anti-C189 antibodies and protein A tagged with 10 nm colloidal gold particles in sequence. All ultrathin sections were sequentially stained with saturated uranyl acetate in 50% ethanol and 0.08% lead citrate. Selected images were observed and photographed under an electron microscope (JEOL JEM-1230, Tokyo, Japan) at 100 kV.

## Results

### Intercellular spread of DENV via cell contact

**Fig 1:**
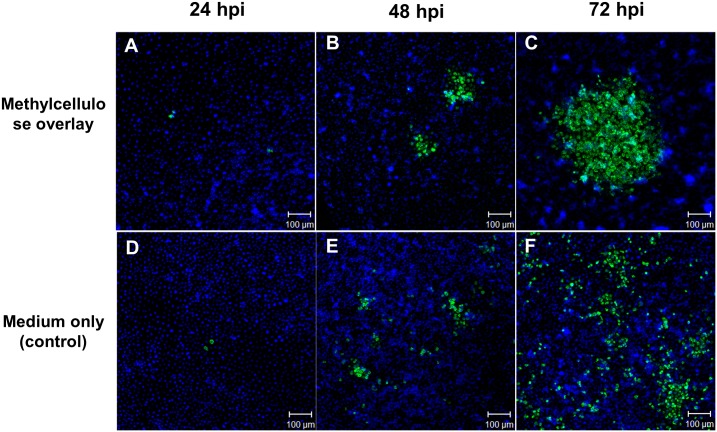
Inference of intercellular spread of DENV by using a 1% methylcellulose-containing overlay. A-C) In the presence of 1% methylcellulose, a few infections appeared in the monolayer of C6/36 cells at 24 hpi while gradually enlarged aggregates of infected cells were observed at 48 and 72 hpi, respectively. D-F) Scattered infected cells are shown in the monolayer of C6/36 cells cultured in the medium only even when the virus was inoculated for 72 h. green: cells positive to antibodies against viral E protein; blue: cells stained with DAPI.

**Fig 2:**
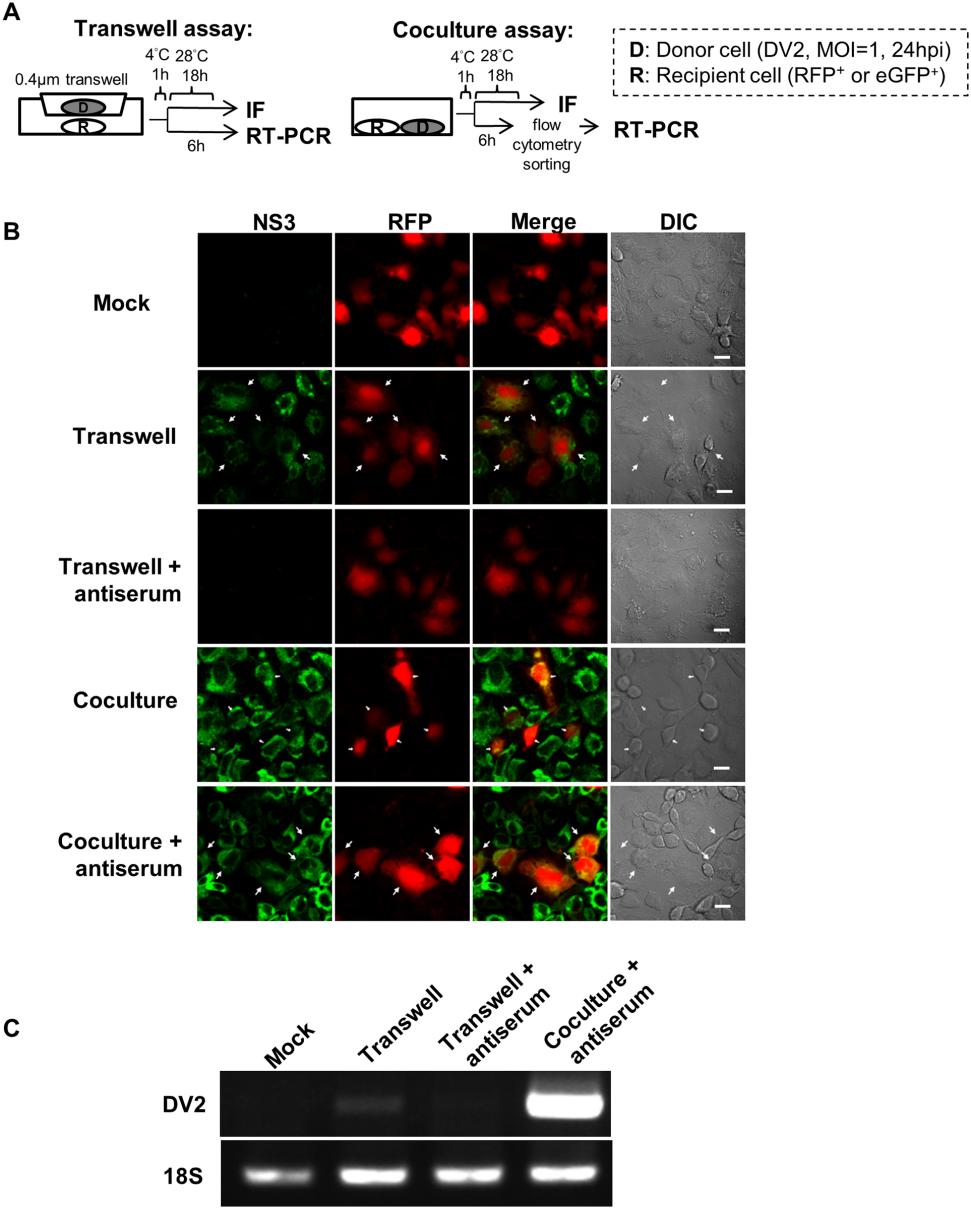
Cell-to-cell transmission of DENV in C6/36 cells demonstrated in transwell and co-culture systems. A) Diagram showing transwell (0.4 μm pore size) or co-culture systems used to investigate virus spread between donor (D) and recipient (R) cells, either with or without the presence of neutralizing antiserum (NeuAb). For more detailed information, please see the description in the Materials and Methods. Cells were observed with an immunofluorescence assay (IFA) after treatment with respective antibodies. B) Mock-infection donor cells were used as controls while recipient cells were transfected with rhodamine fluorescent protein or RFP (red) prior to the investigation. In the transwell system, donor cells infected by DENV for 24 h at MOI = 1 were identified by polyclonal antibodies against the viral NS3 protein (green). Meanwhile, some recipient cells were actually infected by the virus, indicating the possibility of diffuse movement by viruses released by donor cells (white arrow). As no infection occurred in recipient cells in the presence of NeuAb, it seemed that intercellular virus spread may have been blocked by adding NeuAb. Results from co-cultures revealed that a number of recipient cells were infected and thus positive to NS3 antibodies even NeuAb has been added to the culture. This suggested an intercellular spreading mode by DENV, most likely by cell-to-cell transmission. C) Recipient cells sorted by flow cytometry were collected for viral RNA analysis by reverse transcriptase (RT)-PCR. No viral RBA can be detected in mock-infected cells (mock). A small amount of viral RNA was detected in cells collected from the transwell system without NeuAb (transwell), but not in the presence of antiserum (transwell+antiserum). However, a large amount of viral RNA was amplified in the co-culture group even in the presence of NeuAb (co-culture+antiserum), further implying the possibility of cell-to-cell transmission of DENV. The RNA level of 18S was used as the internal control in this experiment.

Observation of the intercellular spread of DENV in C6/36 cells was first carried out by using the monolayer overlaid with 1% methylcellulose-containing semi-solid medium as the conventional plaque assay for virus titration. Infected cells sporadically appeared widely on the monolayer at 24 hpi either with or without a methylcellulose overlay (Fig 1a and 1d). Aggregates of infected cells started to appear on the overlaid monolayer at 48 hpi (Fig 1b and 1e), which became more evident at 72 hpi (Fig 1c and 1f). This implied the occurrence of DENV cell-to-cell transmission under conditions of high cell density even though the possibility of a limited rate of diffusion by released virions could not be completely excluded. Therefore, this phenomenon was further investigated by utilizing transwell or co-culture system methods to confirm the intercellular spread between virus-infected donor and RFP-transfected recipient cells (Fig 2a). A few recipient cells were infected by DENV at 18 hpi in the transwell-containing medium only while no infected recipient cells were observed in the same system in the presence of NeuAb (Fig 2b). On the other hand, most recipient cells became infected at 18 hpi in the co-culture system even when NeuAb was added to the culture medium (Fig 2b). Additionally, RT-PCR revealed that a large copy number of viral RNA was detected only from recipient cells in the co-culture system treated with NeuAb (Fig 2c). Though a low amount of viral RNA was detected in cells from the transwell system without NeuAb treatment, no sign of viral infection appeared in the presence of NeuAb (Fig 2c), further implying that the efficiency of diffuse movement by the virus released from donor cells is relatively low. All results suggested that DENV spreads from one cell to another by direct contact or cell-to-cell transmission.

### Quantitative assessment of DENV cell-to-cell transmission

**Fig 3:**
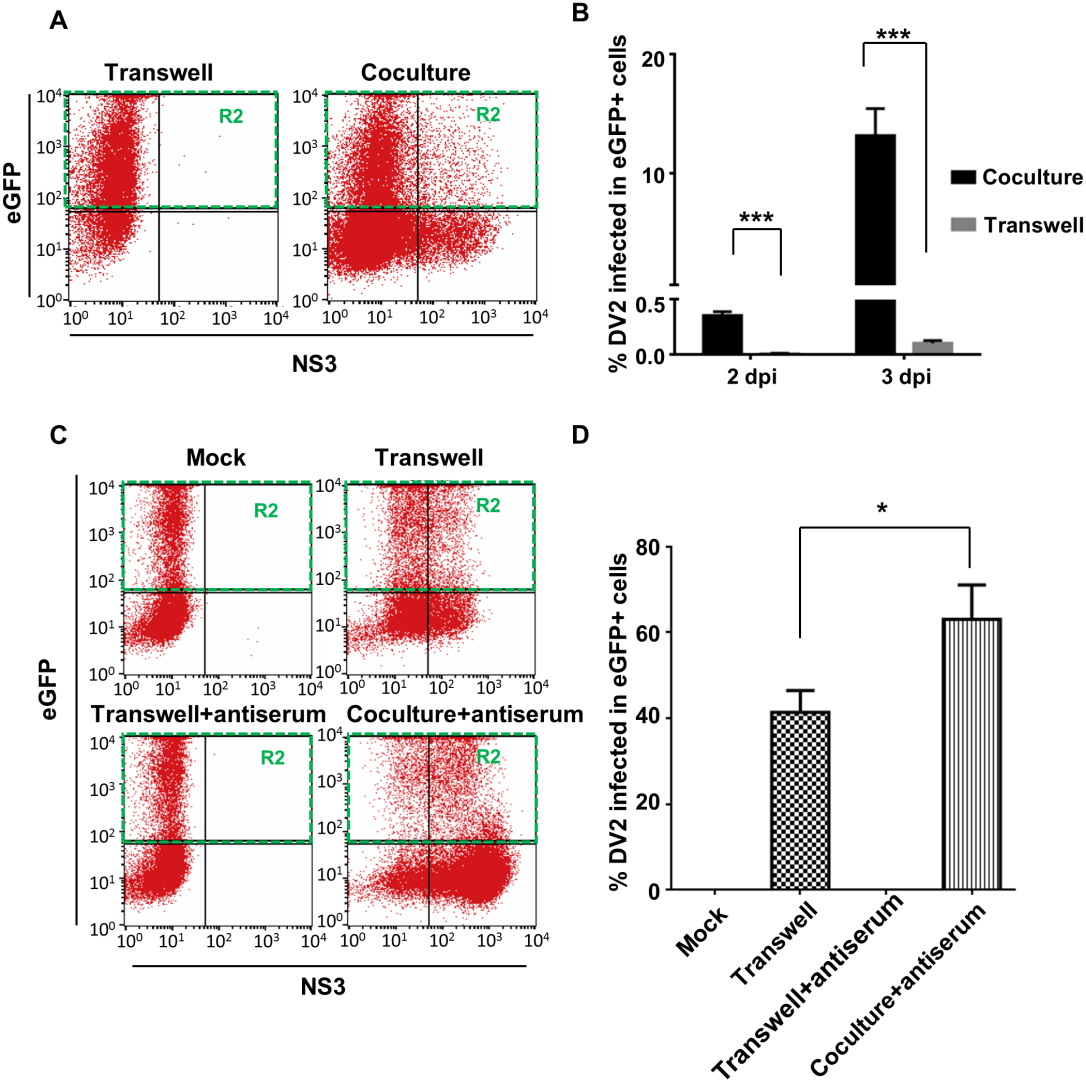
Quantitative assessment for cell-to-cell transmission of DENV. A) Transwell and co-culture systems were further used to quantify cell-to-cell transmission of DENV; the study design followed that shown in the Fig 2A, except for the replacement of RFP by eGFP to label recipient cells. In the experiment, eGFP-transfected recipient cells from both systems at 2 or 3 dpi were gated for detection of viral NS3 protein, which was mostly detected in recipient cells collected from the co-culture system. B) The infection rate of recipient cells from the transwell system was significantly lower than the co-culture system at either time points; *i*.*e*., 2 or 3 dpi (Student’s *t*-test; *p*<0.01). C) Viral NS3 was not shown in cell with mock-infection while a proportion of recipient cells were infected without treatment of NeuAb in the transwell system. Nevertheless, no infected recipient cells appeared in the same system treated with NeuAb, indicating antiserum may restrict the virus diffusion movement. More, recipient cells in the co-culture system in the presence of NeuAb, was still easily infected by the virus due to a large proportion of cells containing viral proteins can be detected. D) Among the two groups containing infected recipient cells, the proportion of cells positive to viral NS3 in the NeuAb-free transwell system (41.40%) was significantly lower (63.02%) than in the co-culture system in the presence of antiserum (Student’s *t*-test, *p*<0.05). Mock infection was used as the negative control in this experiment. It seemed that the intercellular spread of the DENV via cell-to-cell transmission was more efficient than diffuse movement of cell-free viruses.

Co-culture and transwell systems were further used to quantify the cell-to-cell spread of dengue virions in C6/36 cells by flow cytometry. By this quantitative assessment, eGFP-transfected recipient cells from both systems were gated by flow cytometry to detect the existence of viral NS3 antigen (Fig 3a). It was found that less than 0.01% of recipient cells in the transwell system at 2 dpi were infected while infection was 0.35% in the co-culture system. Meanwhile, 0.10% of recipient cells were NS3-positive at 3 dpi, which was significantly lower (13.15%) than for the co-culture system (Student’s *t*-test, *p*<0.01). This provided further evidence that cell contact significantly increased the efficiency of DENV intercellular spread in C6/36 cells. Further investigation was subsequently carried out in the above systems with and without specific neutralizing Abs (NeuAb), revealing that NS3 can be mostly detected in recipient cells from co-culturing (Fig 3b). When NeuAb was applied to prevent diffusion to recipient cells by released virions, no NS3 was detected in cells from the mock-infected co-culture or the lower well in the transwell system (Fig 3c). NS3-positive recipient cells from the transwell system without NeuAb (41.40%) were significantly lower than that in the co-culture system under the same conditions (63.02%) (Student’s *t*-test, *p*<0.05) (Fig 3d). This revealed that cell-to-cell transmission of DENV was more efficient than diffuse movement for cell-free virions released by donor cells.

### Association of DENV-induced C189 up-regulation with virus cell-to-cell transmission in C6/36 cells

**Fig 4:**
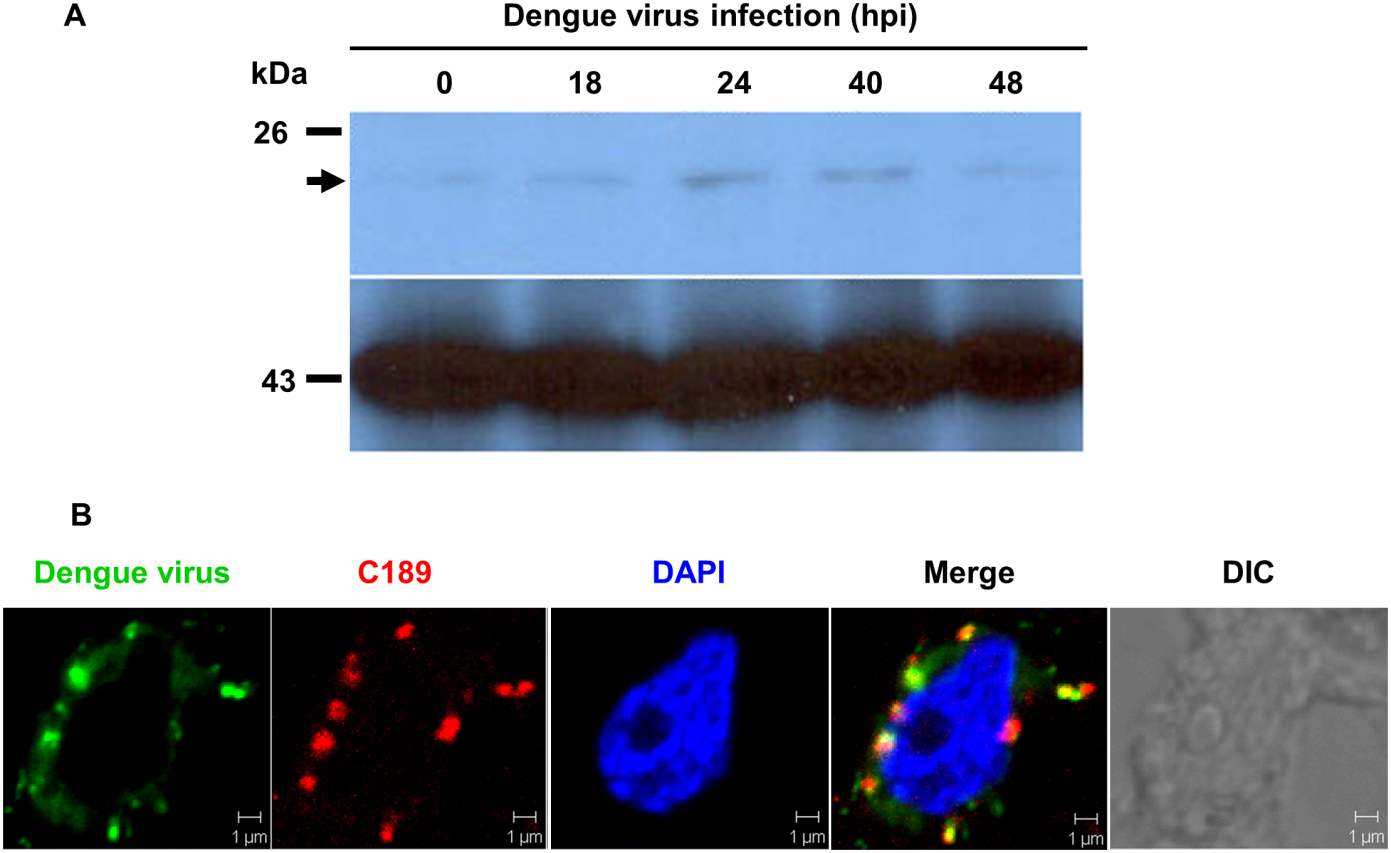
Upregulation of tetraspanin C189 and its association with cell-to-cell transmission in C6/36 cells. A) At the protein level, endogenous C189 was up-regulated at 18 hpi and persisted until 48 hpi in C6/36 cells infected by DENV, consistent with our previous detection of C189 at the RNA level. Actin was used as the internal control in the experiment. B) Under confocal microscopy, C189 appeared as red spots in response to infection by DENV. It was largely co-localized with viral E protein (green) in the cytoplasm of infected cells, implying that they have an intimate association in C6/36 cells infected by DENV.

**Fig 5:**
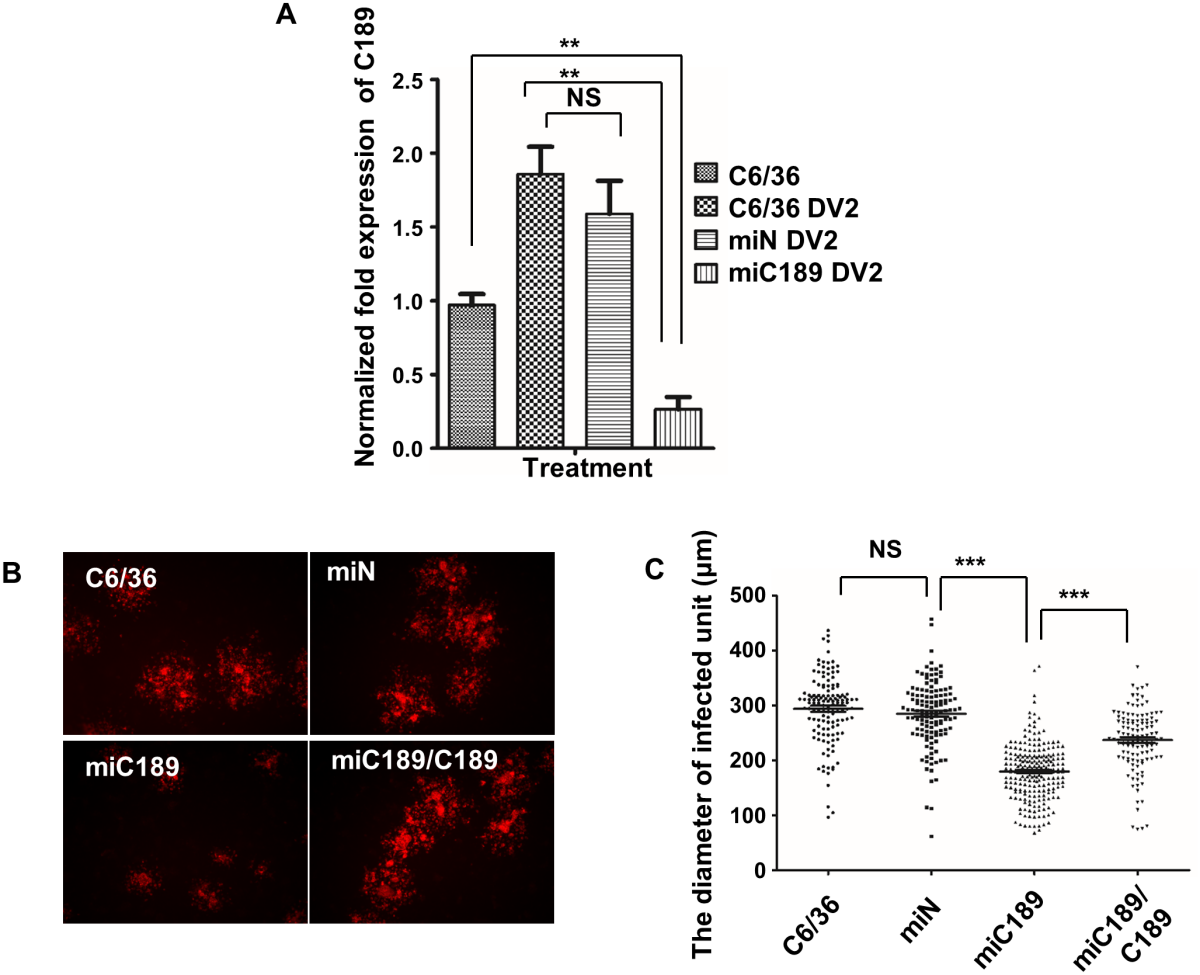
The role of C189 in cell-to-cell transmission of DENV. A) Real-time (RT)-PCR C6/36 cells untransfected or transfected with an unrelated sequence (miN) both increased the expression of C189 in response to DENV infection. On the other hand, its expression level was significantly reduced when C189 was knocked down by a microRNA-based knockdown system (miC189) (Student’s *t*-test, *p*<0.01). B) Based on the overlay of 1% methylcellulose, C189 knockdown restricted the aggregation of infected cells that were either untransfected or transfected with an unrelated sequence. When a recovery system using a C189-overexpressing plasmid was applied to miC189 cells (miC189/C189), an increase in infected cell aggregates appeared. C) The average diameter of aggregated cells in the C189 knockdown group (miC189) was 170 μm, which was significantly smaller than controls, either C6/36 cells (290 μm) or miN (280 μm), and even the recovered group (270 μm) (Student’s *t*-test, *p*<0.05). This indicates that C189 is essential during cell-to-cell transmission of DENV in C6/36 cells.

The RNA level of C189 increases in response to DENV-2 infection in C6/36 cells [29]. This was confirmed herein at the protein level, which increased at 18 hpi and persisting until 48 hpi in infected C6/36 cells (Fig 4a). Imaging using double staining IFA showed that C189-containing spots that were formed in response to DENV-2 infection appeared to co-localize with the viral E protein in the cytoplasm of infected cells at 24 hpi (Fig 4b). This suggested that there is an intimate association between the two proteins during DENV-2 infection in C6/36 cells. To further confirm the role of C189 involved in the cell-to-cell spread of the virus, a microRNA-based system (miC189) to knockdown C189 was constructed and transfected into C6/36 cells with DENV-2 infection for 24 h. This revealed that C189 increased at the RNA level as shown before, while significantly declining to 27.34% and 15.23% in cells of miC189 with mock-infection and—transfection, respectively (Student’s *t*-test, *p*<0.01). It was even reduced to 15.23% when compared with the cell control (Student’s *t*-test, *p*<0.01) (Fig 5a). Our results also showed that the expression of C189 at the RNA level were not significantly different in infected cells with mock-transfection or those transfected with a scramble sequence (miN) (Fig 5a), indicating that no deleterious effect was derived from transfection in this experiment. The effect of C189 in DENV cell-to-cell spread was further evaluated by the overlay of 1% methylcellulose on C6/36 cells. Virus spread was evidently restricted in the miC189 group compared to the two control groups as above while a reverse increase was observed when a recovery system using a C189 overexpressing plasmid was applied to miC189 cells (Fig 5b), suggesting that virus spread was possibly associated with C189. In addition, we have obtained results from the co-culture assay that revealed enhanced efficiency of viral proteins transfer from donor cells to recipient cells but reduced when C189 was knocked down in C6/36 cells (S1 Fig). The diameter of the aggregated cell area in miC189 (170 μm on average) was significantly smaller than in the two control groups (290 μm for the untransfected group and 280 μm for the miN group), even in the group receiving C189 recovery (270 μm) (Student’s *t*-test, *p*<0.05) (Fig 5c). This indicated that C189 might be essential in cell-to-cell DENV spreading in C6/36 cells.

### C189-containing membrane-bound vacuoles are formed in C6/36 cells in response to DENV infection

**Fig 6:**
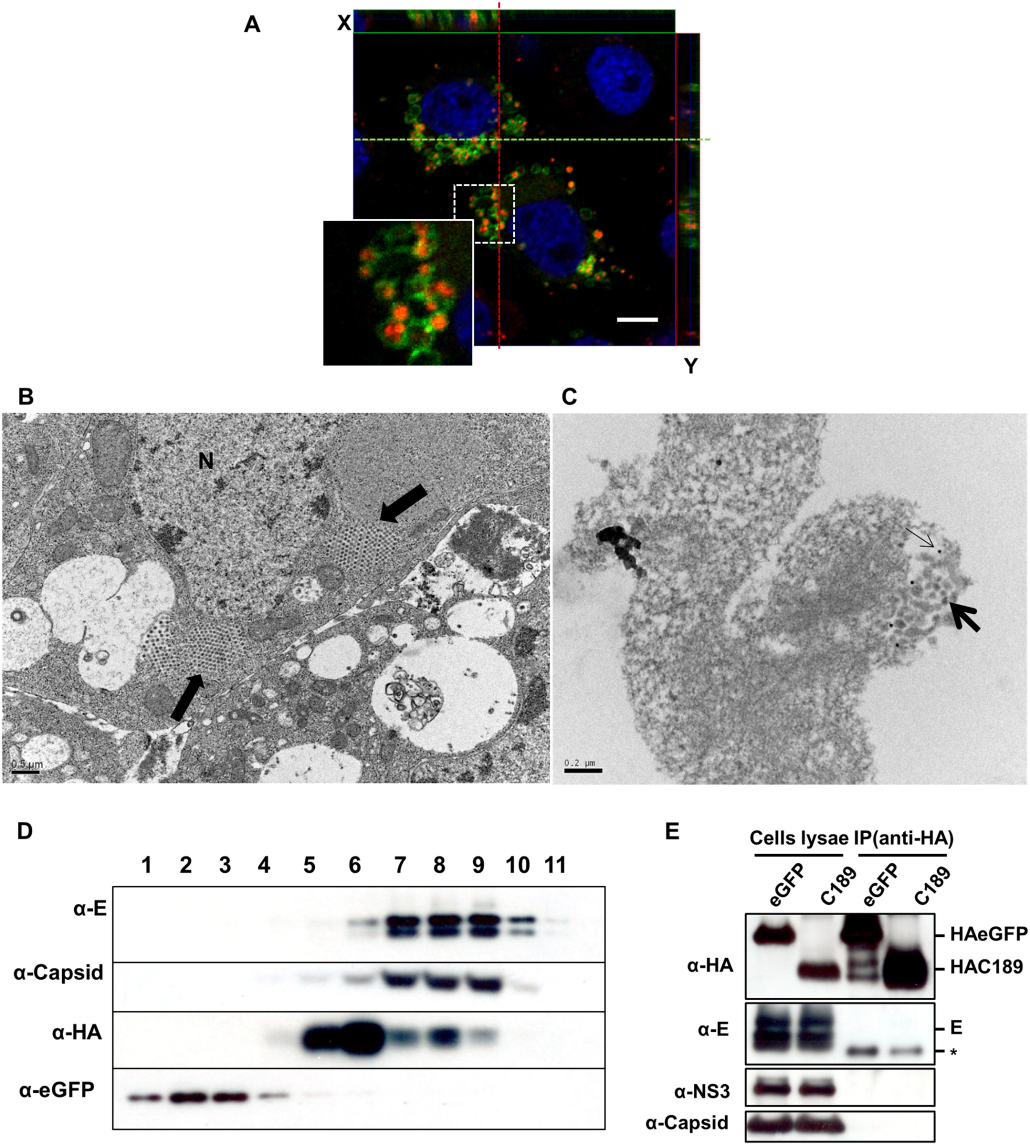
C189-containing membrane-bound vacuoles were formed to deliver DENV via cell-to-cell transmission. A) Confocal microscopy revealed that membrane-bound vacuoles containing C189 (C189-VCs) (green) were formed in DENV-infected C6/36 cells overexpressing eGFP-tagged C189, within which viral E protein (red) or the virus were contained. B) An ultrastructural photograph showing that vacuoles containing numerous virions (arrow) were usually formed in the cytoplasm of C6/36 cells infected by DENV, usually at 24 hpi. C) Immunocytochemical study using colloidal gold particles to label C189 showing vacuoles containing maturing virions (thick arrow) were peripherally positive to the staining of C189 antibodies (thin arrow). This indicated that C189-VCs might be where viral proteins and even virus particles are stored. D) Further sucrose gradient density ultracentrifugation to separate intracellular components from cell lysates of DENV-infected C6/36 cells transfected with HA-tagged plasmid containing the C189 insert (HA-tagged C189) revealed that envelope (E) and capsid (C) proteins of the virus were both detected simultaneously with C189 (represented by positive to antibodies against HA) in fractions containing higher concentrations of sucrose. However, no viral proteins were detected in cell lysate of controls that were transfected with eGFP only. E) Immunoprecipitation (IP) performed with constructed HA-tagged C189 showed that viral proteins (E, NS3, and C) did not directly interact with C189, although a weak band (*) appeared at the position below the E protein. It seemed that C189 in C189-VCs formed in DENV-infected C6/36 cells in response to DENV infection may not have a direct interaction although they coexist spatially in the cell.

The spatial distribution of C189 and viral E protein in DENV2-infected C6/36 cells overexpressing eGFP-tagged C189 was investigated using confocal microscopy with pinhole narrow-down, acquiring more z-axle optical sections thinner than 0.5 μm for further observation on higher resolution images. Interestingly, viral E protein was found to be surrounded within C189-bound vacuoles (C189-VCs) that were formed in the cytoplasm in response to DENV infection in C6/36 cells (Fig 6a). An alternative approach using HAC189 fusion protein (C189 fused with HA-tag at the N-terminal domain) separately applied to DENV-infected cells further supported the viral E protein being localized in C189-VCs (S2 Fig). An ultrastructural study revealed that vacuoles containing numerous virions were usually formed in DENV-infected C6/36 cells, usually at 24 hpi (Fig 6b), which were peripherally positive to staining with anti-C189 antibodies according to the immunocytochemistry study (Fig 6c). This indicated that C189-VCs might be a reservoir not only for viral protein but also for virus particles. In order to clarify the relationship between the viral protein and C189-VCs formed in response to infection, sucrose gradient density ultracentrifugation was used to separate intracellular components from cell lysates of C6/36 cells infected by the virus and subsequently transfected with HA-tagged plasmid containing the C189 insert (HA-tagged C189). No viral protein appeared in the lysate of uninfected cells co-transfected with eGFP- and HAC189-expressing vectors according to Western blot results for proteins from each collected fraction. However, envelope (E) and capsid (C) proteins of the virus and C189 (positive to HA) were simultaneously detected in fractions with higher concentrations of sucrose (Fig 6d). In further investigation through immunoprecipitation (IP) performed with HA-tagged C189, none of the three viral proteins (E, NS3, and C) directly interacted with C189, although a weak band occurred close to the position of the E protein (Fig 6e). One possibility of this weak band reflects the heavy chain of mouse anti-HA monoclonal antibody used in this study as mentioned elsewhere [33]. These observations revealed that C189-VCs were usually formed in C6/36 cells in response to DENV infection, during which C189 spatially coexists with viral proteins or virions. However, there might be no direct binding occurring between them.

### Cell-to-cell DENV transmission via C189-containing membrane-bound vacuoles

**Fig 7:**
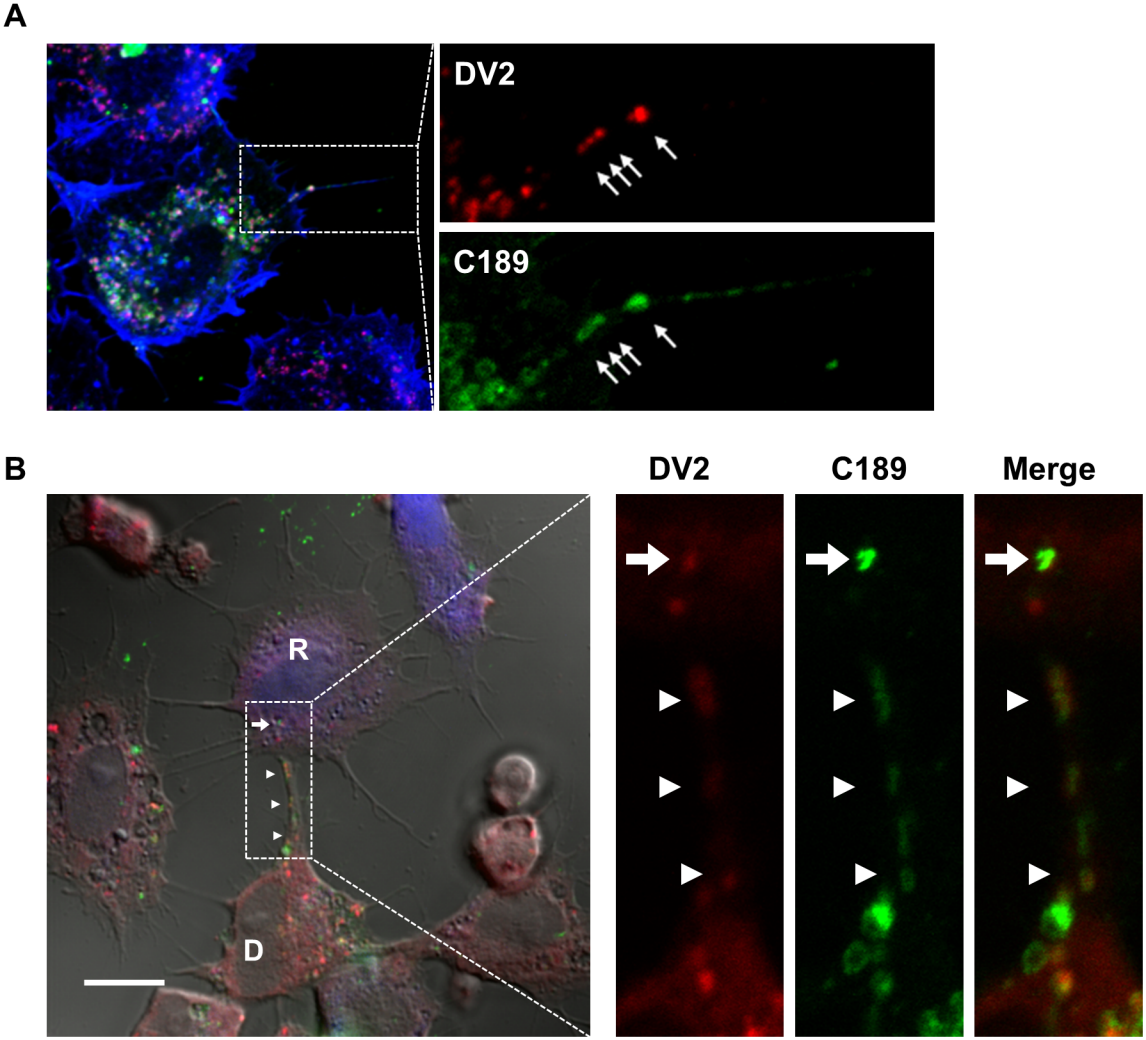
Intercellular translocation of C189-containing membrane-bound vacuoles containing DENV in C6/36 cells. A) In culture for C189eGFP-expressing cells infected by DENV2, viral E protein (red and arrow) co-localizes with C189 (green and arrow) at 24 hpi, which is frequently seen in filopodia extended by donor cells. B) For further clarification, C189eGFP-expressing donor cells infected by DENV were co-cultured with RFP-expressing recipient cells in the presence of neutralizing antibodies (NeuAb) for 6 h. It turns out that viral E protein and C189 were still delivered simultaneously from the donor cell to the recipient cell (arrow) via filopodia (arrow head). D: the donor cell; R: the recipient cell. Scare bar = 10 μm.

In culture for C189eGFP-expressing cells infected by DENV2, viral E protein was usually seen to co-localize with C189 at 24 hpi, which were frequently seen filopodia extended from donor cells (Fig 7a). The filopodia usually touched the recipient cell; through the contact site virus-containing C189 CVs were thus delivered to neighboring recipient cells (S1 Movie). For clarification, C189eGFP-expressing donor cells infected by DENV-2 were further co-cultured with RFP-expressing recipient cells for no longer than 6 h in the presence of NeuAb. Observations on the distribution of viral proteins and C189 by confocal microscopy showed that both the viral E protein and C189 were simultaneously detected in filopodia and were delivered to recipient cells even when cell-free virions have been blocked to release into the medium by treatment with antiserum (Fig 7b).

## Discussion

Arboviruses naturally infect vertebrate hosts through biting by hematophagous arthropod vectors, requiring the ability to replicate in the cells of both hosts. Like most arboviruses in humans, free DENVs are usually released into the circulatory system and subsequently infect susceptible cells via endocytosis, which are usually receptor(s)-mediated and clathrin-dependent [34, 35]. DENVs can also infect mosquito cells via a mode similar to that which occurs in vertebrate cells [36], although their specific receptors have not yet been clearly identified [13]. DENV dissemination within the mosquito has recently attracted increased attention. It seems that DENV-2 can infect various organs (*e*.*g*., neural tissue and salivary glands [20]) disseminating from the midgut, which is the original lodging and replication site of the virus when ingested along with blood meals [37]. The virus travels from the midgut to salivary glands before being transmitted to another host.

The virus probably does not depend highly on the release-and-entry mode due to the close proximity of epithelial cells within a tissue such as the midgut in the vector host [18]. Since mosquito cells do not die from DENV infection [15, 16], the release-and-infect mode of extending infection into mosquito tissues is less likely. In the present study, intercellular DENV spread in C6/36 cells was first detected by limiting the diffusion of released viruses within methylcellulose-containing medium. As the aggregation of infected cells became more evident, it brought up the possibility of DENVs being transmitted to neighboring cells by a cell-to-cell mode. A similar result also occurred in assays with transwell and co-culture systems, even when NeuAb was applied to neutralize released virions to eliminate the effect of diffusion. Recipient cells were more easily infected by donor cells infected with DENVs, especially when the possibility of contact between the two types of cells was high.

Increasing evidence reveals that specific host genes may be up-regulated, playing important roles in regulating viral infection and corresponding responses by infected cells [38]. The gene effects may cover most steps of the infection, from the viral entry, RNA replication, as well as assembly and release of progeny virions [39]. For instance, a total of 305 host proteins have been identified from human cells infected by West Nile virus, suggesting a close association between the virus and host cells [40]. Some of the identified host factors are important in cell-to-cell transmission of the virus [28]. In fact, the novel tetraspanin C189 from C6/36 cells is usually up-regulated in response to DENV-2 infection and is suspected to associate with the intercellular spread of the virus among mosquito cells [29]. Tetraspanins such as CD9 and CD81 play a role in modulating cell-to-cell transmission during HIV infection [41], consistent with our theory [29]. In this study, we have further observed co-localized endogenous C189 and viral E protein in C6/36 cells. When C6/36 cells were transfected with a C189-expressing construct, highly expressed C189 was incorporated into the membrane of virus-responsive vacuoles, called C189-containing membrane-bound vacuoles (C189-VCs), within which virions and/or viral proteins were confined. This further implicates the importance of tetraspanin involvement in the intercellular spread of arboviruses such as DENVs in mosquito cells. It is consistent with some extracellular vesicles that contain clusters of tetraspanins in their membranes to form microdomains [42].

Tetraspanins naturally existing in a broad spectrum of organisms possesses various biological functions including the regulation of cell proliferation, cell-cell or cell-extracellular matrix interactions, and cell motility [43]. They may also be involved in promoting metastasis in carcinomas [44] and act as transmembrane linkers [45, 46]. Further, tetraspanins were found to be involved in different kinds of microbial infections [47] such as T lymphocyte infection by HIV-1 and HTLV [48]. More specifically, in hepatitis C virus (HCV) infection of hepatocytes, CD81may serve as a receptor or co-receptor presumably via its association with the viral E2 protein [49, 50]. Nevertheless, according to the present study, C189 may not serve as the receptor of C6/36 cells during infection by DENVs as virus production evidently did not change in C6/36 cells with knockdown of C189 and/or further recovery by overexpression of the same protein. It seems that this tetraspanin more likely participates in intercellular transmission of C189-VCs formed in cell cytoplasm, which is supposed to be beneficial for virus delivery between C6/36 cells having resistance to host defense as in hepatocytes infected by HCV [50, 51]. To confirm the specific role of C189 involvement in the intercellular transmission of associated vacuoles, C6/36 cells were over-expressed with a panel of different proteins, including tetraspanins C189 and ER-related proteins including GRP94/endoplasmin and GRP78/BiP, as well as controls containing only eGFP (S3 Text and S3 Fig). Among these, only C189 was efficiently transferred from donor cells to recipient cells, revealing the essentialness of C189 on this process. Since C189 overexpression did not cause significant apoptosis in transfected cells, there was no possibility that artifacts originating from apoptotic bodies were engulfed by recipient cells [52]. C189 overexpressed in uninfected C6/36 cells can also translocate to neighboring cells, indicating that transmission is relatively dependent on cell-cell contact. In a culture containing a lower density of C6/36 cells, C189-VCs with or without virions were also delivered to neighboring cells along filopodia extended by the cell (S1 Movie). As cell death usually does not occur in mosquito cells infected by DENVs, at least for certain strains [17], release-and-entry may not be a normal way for viruses to spread in infected tissues of those cases. As a result, cell-to-cell transmission can be a direct mode for infecting neighboring cells [22]. Generally, tetraspanin proteins are able to mediate cellular penetration, invasion, and fusion events and define a membrane microdomain [53]. They may also modulate virus-induced membrane fusion [41]. In fact, HIV and perhaps also HCV launch cell-to-cell transmission by way of cell contact, in which the tetraspanin CD81 is believed to be involved [26, 28].

In addition to endogenous C189, we have also extensively used fluorescence-tagged C189 as a proxy in this study. Although it is not a completely natural condition, this approach provided convenience and advantages in observing how C189 is associated with DENV, particularly the intercellular spread of the virus. Taken together, DENV generally establishes infections through a release-and-entry mode in cultures, while they are more likely to employ cell-to-cell transmission as an alternative route for spreading progeny virus particles [54]. Tetraspanin C189 was up-regulated and incorporated into the membrane of virus-induced vacuoles, which are presumed to become rapid and efficient vehicles in the delivery of the virus from one cell to another. Our findings are interesting and may be responsible for lateral spread of DENV within the same tissue in a mosquito, and further understanding of its mechanism would increase our ability to intervene in the spread of DENV infections.

## Supporting Information

S1 Fig The efficiency of C189-dependent translocation of membrane protein-bound vacuoles between C6/36 cells. The co-culture system was used to measure the translocation efficiency of membrane-bound vacuoles between C6/36 cells. When donor cells were separately transfected with constructs expressing selected ER-related membrane proteins (*i*.*e*., C189, endoplasmin/GRP94, and Bip/GRP78), only C189-incorporated vacuoles (28.17%) were efficiently transferred into recipient cells. On the other hand, only 0.45% and 0.51% of recipient cells received any structures containing endoplasmin/GRP94 and Bip/GRP78, respectively, from donor cells. No apoptosis occurred in cells transfected with the construct expressing C189, suggesting that fluorescent-positive C189 detected in recipient cells was not likely derived from engulfing apoptotic bodies of transfected donor cells. As a result, C189 is more important and may be an essential molecule involved in translocating membrane-bound vacuoles either with or without virions between cells.
(TIF)

S2 Fig An alternative approach using HAC189 fusion protein (C189 fused with HA-tag at the N-terminal domain) separately applied to DENV-infected cells further supported the viral E protein being localized in C189-VCs. (TIF)

S3 Fig The efficiency of C189-dependent translocation of membrane protein-bound vacuoles in C6/36 cells. The co-culture system was used to measure the translocation efficiency of membrane-bound vacuoles between C6/36 cells. When donor cells were separately transfected with constructs expressing selected ER-related membrane proteins (*i*.*e*., C189, endoplasmin/GRP94, and Bip/GRP78), only C189-incorporated vacuoles (28.17%) were efficiently transferred into recipient cells. On the other hand, only 0.45% and 0.51% of recipient cells received any structures containing endoplasmin/GRP94 and Bip/GRP78, respectively, from donor cells. No apoptosis occurred in cells transfected with the construct expressing C189, suggesting that fluorescent-positive C189 detected in recipient cells was not likely derived from engulfing apoptotic bodies of transfected donor cells. As a result, C189 is more important and may be an essential molecule involved in translocation of membrane-bound vacuoles either with or without virions between cells.
(TIF)

S1 Movie Donor cells in the co-culture system extending filopodia to touch recipient cells. Either C189 CVs-containing viral proteins or the virus itself can translocate to the neighboring cell at the contact site.
(MP4)

S1 Text Production and assay of polyclonal antibodies against C189 LEL (large extracellular loop). (DOCX)

S2 Text A schematic process showing a stable knockdown system establishment used for reduced expression of mosquito genes. (A) Design of miRNA-based stable knockdown vector for inhibition of the C189 gene. (B) The predicted pre-miRNA sequence structure of miC189.
(DOCX)

S3 Text Construction of vectors expressing selected membrane proteins and assay for translocation of membrane-bound vacuoles between cells. (DOCX)

S1 Table The list of primer pairs used for constructs in the related experiments. (DOCX)
