# Supplementary figures and images for: Involvement of Tetraspanin C189 in Cell-to-Cell Spreading of the Dengue Virus in C6/36 Cells
Source: PLoS Negl Trop Dis. 2015 Jul 1;9(7):e0003885. doi: 10.1371/journal.pntd.0003885 (PMC4488468; doi:10.1371/journal.pntd.0003885)

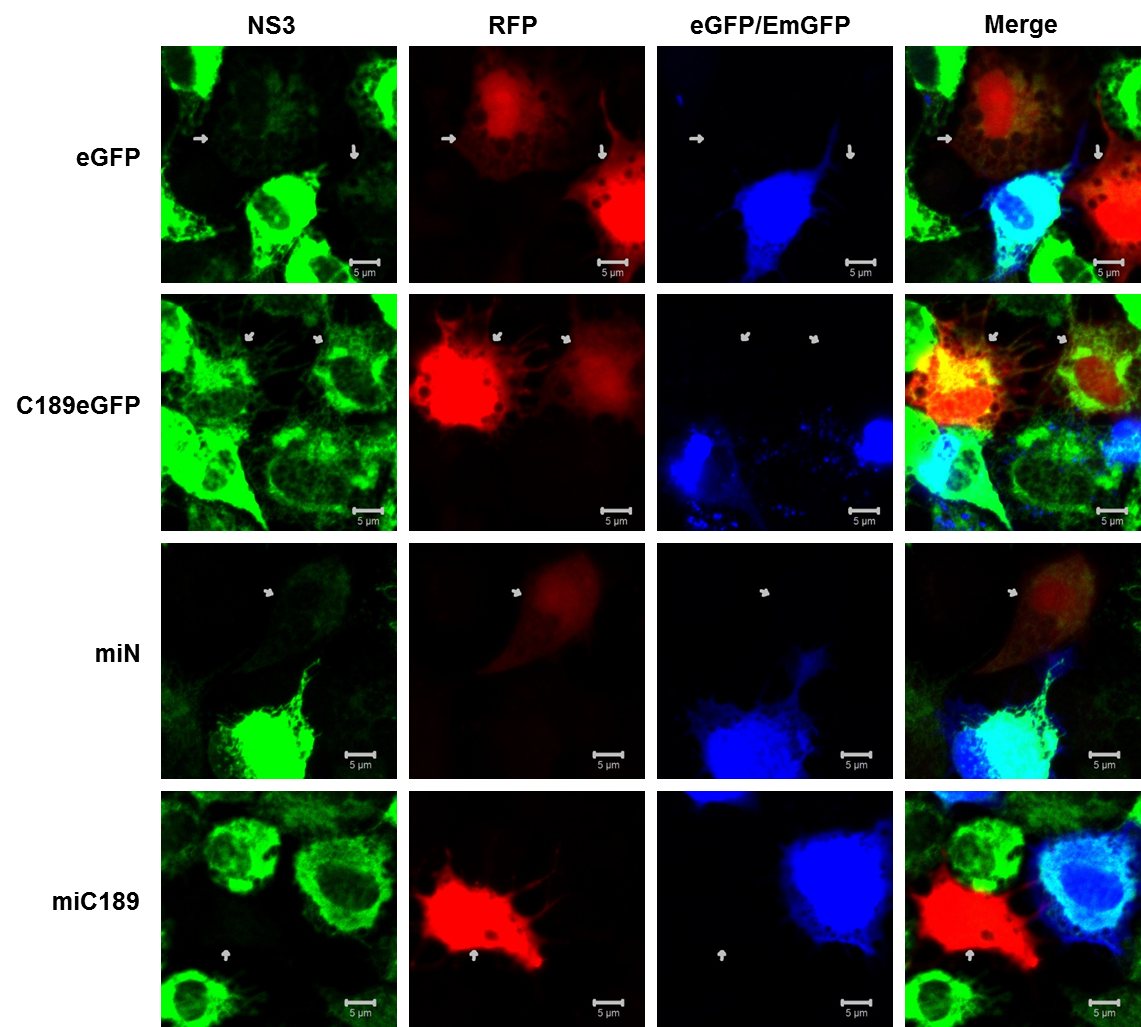

Supplement: S1 Fig — The co-culture system was used to measure the translocation efficiency of membrane-bound vacuoles between C6/36 cells. When donor cells were separately transfected with constructs expressing selected ER-related membrane proteins (i.e., C189, endoplasmin/GRP94, and Bip/GRP78), only C189-incorporated vacuoles (28.17%) were efficiently transferred into recipient cells. On the other hand, only 0.45% and 0.51% of recipient cells received any structures containing endoplasmin/GRP94 and Bip/GRP78, respectively, from donor cells. No apoptosis occurred in cells transfected with the construct expressing C189, suggesting that fluorescent-positive C189 detected in recipient cells was not likely derived from engulfing apoptotic bodies of transfected donor cells. As a result, C189 is more important and may be an essential molecule involved in translocating membrane-bound vacuoles either with or without virions between cells. (TIF) [file pntd.0003885.s001.tif]

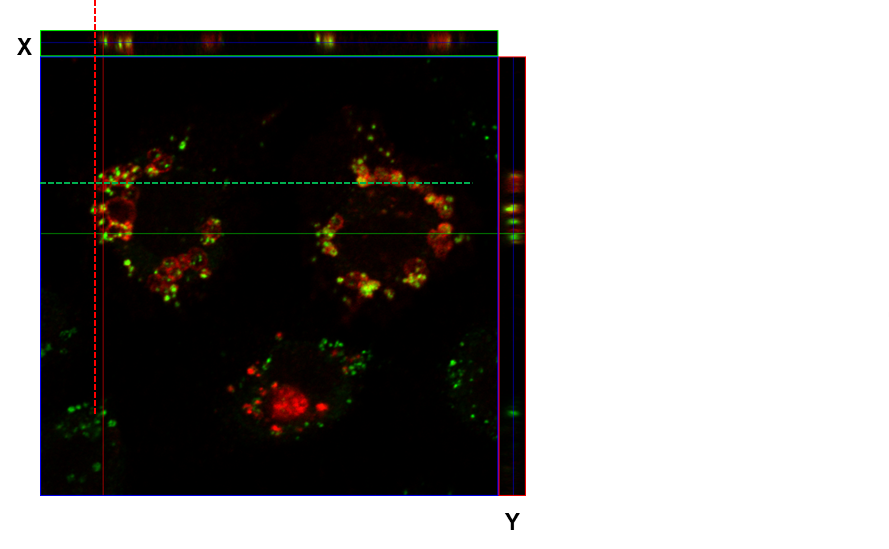

Supplement: S2 Fig — (TIF) [file pntd.0003885.s002.tif]

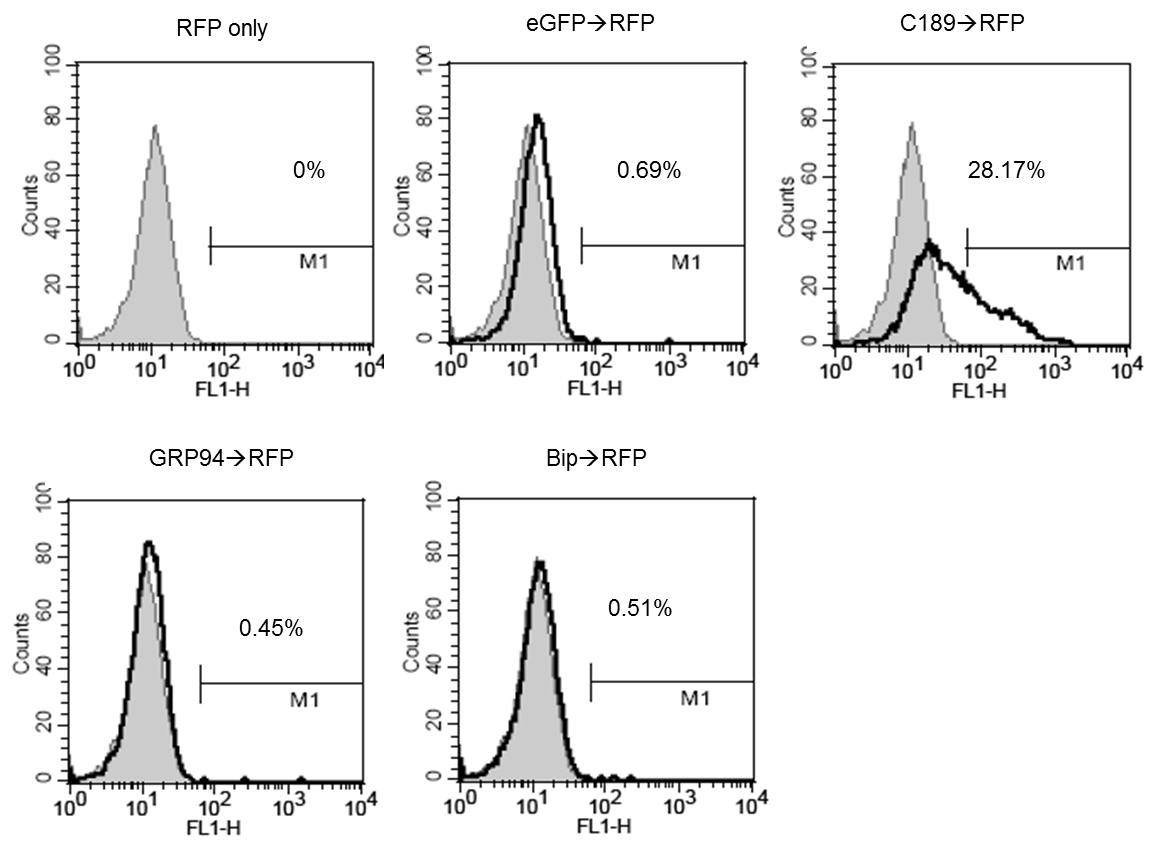

Supplement: S3 Fig — The co-culture system was used to measure the translocation efficiency of membrane-bound vacuoles between C6/36 cells. When donor cells were separately transfected with constructs expressing selected ER-related membrane proteins (i.e., C189, endoplasmin/GRP94, and Bip/GRP78), only C189-incorporated vacuoles (28.17%) were efficiently transferred into recipient cells. On the other hand, only 0.45% and 0.51% of recipient cells received any structures containing endoplasmin/GRP94 and Bip/GRP78, respectively, from donor cells. No apoptosis occurred in cells transfected with the construct expressing C189, suggesting that fluorescent-positive C189 detected in recipient cells was not likely derived from engulfing apoptotic bodies of transfected donor cells. As a result, C189 is more important and may be an essential molecule involved in translocation of membrane-bound vacuoles either with or without virions between cells. (TIF) [file pntd.0003885.s003.tif]
