# Supplementary material for: Involvement of Tetraspanin C189 in Cell-to-Cell Spreading of the Dengue Virus in C6/36 Cells
Source: PLoS Negl Trop Dis. 2015 Jul 1;9(7):e0003885. doi: 10.1371/journal.pntd.0003885 (PMC4488468; doi:10.1371/journal.pntd.0003885)
Supplement: S1 Text — (DOCX) [file pntd.0003885.s005.docx]

**S1 Text.** Production and assay of polyclonal antibodies against C189 LEL (large extracellular loop).

**Cloning of C189 LEL.** The cloning mixture contained 4.5 μl synthesized cDNA, 1 μl each of degenerate primers of 5-GAT ATC GAT GCC AAG GCG GCC TTC (sense) and 5-GGA TCC TCA TAC GTC TCC GGC GGT GTC (antisense), 1 μl 10 mM dNTP, 5 μl PCR buffer, 0.5 μl Ber Taq, and 37 μl H_2_O. The reaction for RT-PCR was run for one cycle at 94 ℃ for 2 min followed by 40 cycles at 94 ℃ for 1 min, 45 ℃ for 1 min, 72 ℃ for 2.5 min, and finally extended for 10 min at 72 ℃. Sense primers had an *EcoR*V site at the 5’ end while the antisense had a *BamH*I site at the 3’ end. A 1983-bp fragment amplified by PCR used extracted total RNA from C6/36 cells as the template [29]. The fragment was then cloned into the pGEM-T vector and subsequently sequenced to confirm its homology to C189 LEL. Subsequent C189 LEL expression and production of the corresponding polyclonal antibodies are described in supplementary materials.

**Expression of C189 LEL.** Plasmid containing the open reading frame of C189 LEL was digested with *EcoR*V and *BamH*I, and a DNA fragment containing the complete open reading frame of C189 LEL cDNA was separated by 0.8% agarose gel electrophoresis. DNA fragments were extracted from the gel and purified with a gel extraction miniprep kit (Viogene, Sunnyvale, CA, USA). The DNA purified from the band on the gel was subcloned into a pET30a (Novagen, Madison, WI, USA) expression vector to prepare the recombinant C189 LEL in *E. coli* strain BL-21 based on the protocol for the pET30 Expression System (Novagen). The inserted DNA fragment was flanked on His-tag and S-tag within the vector. The molecular weight of the expressed fusion protein was estimated to be 78 kDa.

**Production of polyclonal antibodies.** Initial doses of 2 mg of fusion protein were administered in 1 ml Freund’s complete adjuvant (Sigma) to immunize rabbits, followed by a booster with equal quantities of fusion protein in Freund’s incomplete adjuvant (Sigma) at an interval of four weeks. Animals were bled two weeks after the final injection of the antigen. Antiserum, including pre-immunized serum, was collected and tested for efficacy by Western blot.

**Protein extraction from C6/36 cells.** A total of 1 x 10^7^ C6/36 cells were transferred into an Eppendorf tube containing 30 μl RIPA buffer (50 mM Tris-HCl, 140 mM NaCl, 1% Nonidet P40, 0.1% SDS, 0.5% sodium deoxycholate, 100 mM PMSF), homogenized, and then centrifuged at 4 ℃ and 12,000 rpm (14,000 *g*) for 15 min. The supernatant was subsequently transferred into another Eppendorf tube containing an equal volume of 2x sample buffer (0.125 M Tris-HCl, pH 6.8, 4% SDS, 20% glycerol, and 2% 2-mercaptoethanol).

**SDS-PAGE and Western blot analysis.** Purified fusion protein was boiled for 3 min and then separated through sodium dodecylsulfate-polyacrylamide gel electrophoresis (SDS-PAGE) performed on 10% acrylamide gels that were then stained in Coomassie Blue solution. After electrophoresis, unstained samples were transferred from the gel onto Hybond ECL (Amersham Biosciences, Björkgatan, Sweden) nitrocellulose membranes at 60 mA for 4 h. The membrane was blocked with 0.5% non-fat milk and then reacted with anti-C189 LEL serum produced in the rabbit at a dilution of 1:20,000. After washing with PBS, donkey anti-rabbit IgG horseradish peroxidase conjugate (Amersham Biosciences) was added to the membrane at the same dilution. After the reaction, the membrane was exposed to χ-film using a Western Lightning system (PerkinElmer).

**S1 Table. The list of primer pairs used for constructs in the related experiments.**

|  |  |  | |
| --- | --- | --- | --- |
| **Primer** | **Orientation** | **Sequences, 5'-3'** |  |
| **189exF-SalI** | forward | AGGGGTCGACCATGGCACTGAATTGTGGATTATC |  |
| **189exR-NotI** | reverse | ATAGTTTAGCGGCCGCTCAAGCTTAAGCGTATCTTCTC |  |
| **189F2** | forward | GCGCATCGAGAGGGAAAG |  |
| **189R5** | reverse | GTGGAATCAACGCATACCAATG |  |
| **anti-189F** | forward | AAAGATATCATGGCACTGAATTGTGGAC |  |
| **anti-189R** | reverse | AAAGGATCCTCAAGCGTCAACATCAAGC |  |
| **189-F-KpnI** | forward | ATAGGTACCATGGCACTGAATTGTGGATTATC |  |
| **189-R-KpnI** | reverse | TATGGTACCTTAGCGTATCTTCTCTGTTGATTG |  |
| **C189-243 F** | forward | CTGCATGACCACGACCTATGG |  |
| **C189-311 R** | reverse | AGAGCGGCAACGACGATTT |  |
| **EcoRV-HAC189-F** | forward | AAAGATATCAATGGCACTGAATTGTGGATTATC |  |
| **HAC189a-XhoI-R** | reverse | TTTCTCGAGTTAAGCGTATCTTCTCTGTTGATTG |  |
| **miC189-83 bottom** | forward | CCTGAAACGCCTCCGTACCACCAGTCAGTCAGTGGCCAAAACTGGTGGTAATCGGAGGCGTTTC |  |
| **miC189-83 top** | reverse | TGCTGAAACGCCTCCGATTACCACCAGTTTTGGCCACTGACTGACTGGTGGTACGGAGGCGTTT |  |
| **EcoRI-mi-F** | forward | AAAGAATTCCTAGTTAAGCTATCAACAAGTTTG |  |
| **mi-R-Not I** | reverse | TTTGCGGCCGCATCAACCACTTTGTACAAGAAAG |  |
| **eGFP-F-KpnI** | forward | CGGGGTACCATGGTGAGCAAGGGCGAGG |  |
| **eGFP-R-NotI** | reverse | TTTGCGGCCGCTTACTTGTACAGCTCGTCC |  |
| **XhoI-eGFP-F** | forward | AAACTCGAGATGGTGAGCAAGGGCGAGGA |  |
| **eGFP-ApaI-R** | reverse | AAAGGGCCCTTACTTGTACAGCTCGTCCATG |  |
| **EcoRV-HAeGFP-F** | forward | AAAGATATCAATGGTGAGCAAGGGCGAGG |  |
| **HAeGFP-XhoI-R** | reverse | TTTCTCGAGTTACTTGTACAGCTCGTCCATG |  |
| **EcoRI-RFP-F** | forward | AAAGAATTCATGGTGTCTAAGGGCGAAGAG |  |
| **RFP-NotI-R** | reverse | TTTGCGGCCGCTTAATTAAGTTTGTGCCCCAGTTTG |  |
| **Q18SF** | forward | AGGTCCGTGATGCCCTTAGA |  |
| **Q18SR** | reverse | TACAATGTGCGCAGCAACG |  |
| **18SF** | forward | TGACTCAACACGGGAAAAC |  |
| **18SR** | reverse | CAGAACATCTAAGGGCATCAC |  |
| **KpnI-HA-EcoRI-F** | forward | CATGTACCCATACGATGTTCCAGATTACGCTCG |  |
| **KpnI-HA-EcoRI-R** | reverse | AATTCGAGCGTAATCTGGAACATCGTATGGGTACATGGTAC |  |
| **GRP94-EcoRV-F** | forward | AAAGATATCATGAAGTACCTGCTGCTTCTG |  |
| **GRP94-∆HDEL-HA-NotI-R** | reverse | AAAGCGGCCGCAAAGCGTAGTCTGGGACGTCGTATGGGTACTCGGCATCATCGTCGG |  |
| **Bip-EcoRV-F** | forward | AAAGATATCATGAAGCTGCTAGTACCGTTGGCCC |  |
| **Bip-∆KDEL-HA-NotI-R** | reverse | AAAGCGGCCGCAAAGCGTAGTCTGGGACGTCGTATGGGTAGAGATCGTCATCTTCGCCGGCAG |  |
