# Supplementary material for: Involvement of Tetraspanin C189 in Cell-to-Cell Spreading of the Dengue Virus in C6/36 Cells
Source: PLoS Negl Trop Dis. 2015 Jul 1;9(7):e0003885. doi: 10.1371/journal.pntd.0003885 (PMC4488468; doi:10.1371/journal.pntd.0003885)
Supplement: S2 Text — (A) Design of miRNA-based stable knockdown vector for inhibition of the C189 gene. (B) The predicted pre-miRNA sequence structure of miC189. (DOCX) [file pntd.0003885.s006.docx]

**S2 Text.** A schematic process showing a stable knockdown system establishment used for reduced expression of mosquito genes. (A) Design of miRNA-based stable knockdown vector for inhibition of the C189 gene. (B) The predicted pre-miRNA sequence structure of miC189.


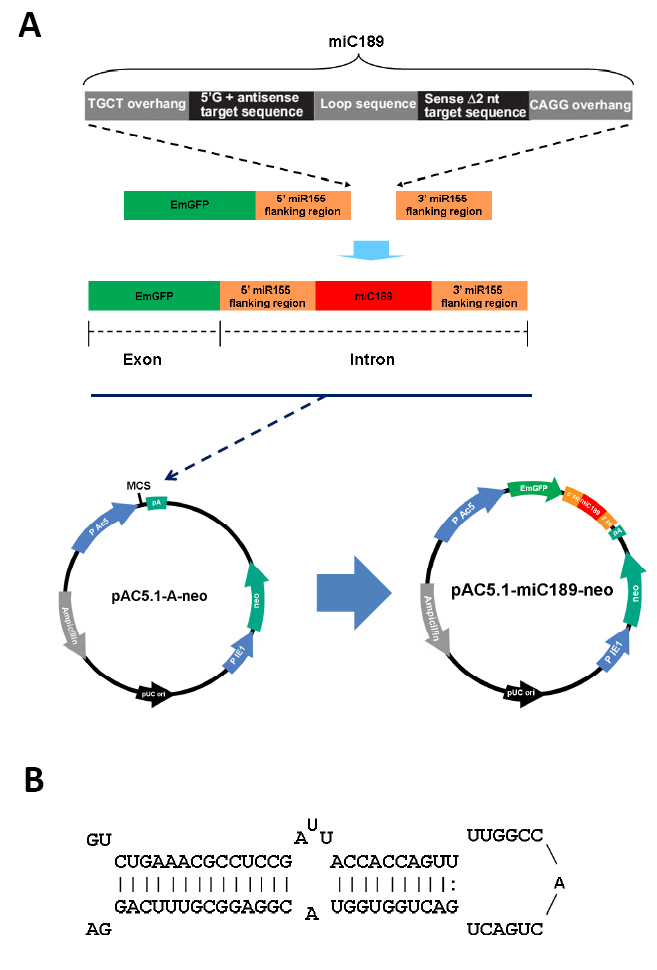


**A**

**B**
