# Supplementary material for: Involvement of Tetraspanin C189 in Cell-to-Cell Spreading of the Dengue Virus in C6/36 Cells
Source: PLoS Negl Trop Dis. 2015 Jul 1;9(7):e0003885. doi: 10.1371/journal.pntd.0003885 (PMC4488468; doi:10.1371/journal.pntd.0003885)
Supplement: S3 Text — (DOCX) [file pntd.0003885.s007.docx]

**S3 Text.** Construction of vectors expressing selected membrane proteins and assay for translocation of membrane-bound vacuoles between cells.

**Construction and transfection of expression vectors in C6/36 cells.** Expression vectors were based on insect-cell-expression vector pAC5.1-V5-His A (Invitrogen). For expressing eGFP fusion proteins, the open reading frame of C189, Bip, and GRP94 amplified by PCR used the indicated primers and template (Supplementary Tables 1, 2) and were inserted into pAC5.1-eGFP expression vector in the N terminal domain of eGFP. In Bip and GRP94-expressing vectors, ER signal peptides were shifted to the C terminal domain of eGFP. To express red fluorescent protein (RFP), the RFP gene was amplified from pTagRFP-C (Evrogen, Moscow, Russia) and inserted into pAC5.1-V5-His A. To express HAeGFP and HAC189, primers HA-F and HA-R were hybridized and ligated with pAC5.1-V5-His A to generate pAC5.1-HA. eGFP and C189 genes were amplified using the indicated primers and template (Supplementary Tables S1) and then inserted into pAC5.1-HA to form HAeGFP and HAC189 expression vectors. For transfection, C6/36 cells were seeded in 6-well plates and grown to 70-80% confluence. X-tremeGene HP DNA transfection reagent (Roche) was mixed with vectors (ratio = 3:1 μl/μg, 1 μg plasmid DNA per well was used in most experiments) in basal medium (MEM, 2% non-essential amino acid, 0.0375% sodium bicarbonate, 0.2% HEPES) at room temperature (RT) for 15 min. Cells were incubated with transfection mixture for 5 h before replacing with complete medium.

**Assay for translocation of membrane proteins between cells.** Co-culture and transwell assays were both implemented in this part of the study. In the assay implemented in this part of the study, C6/36 cells were cultured in 6-well plates until about 80% of the monolayer was formed. Cells in wells were then transfected with pAC5.1-RFP to serve as recipient cells while those transfected with plasmids (including pAC5.1-eGFP, pAC5.1-C189eGFP, pAC5.1-GRP94eGFP, and pAC5.1-BipeGFP) were used as donor cells. All transfected cells were washed three times with PBS and then trypsinized, from which each type of donor cells were co-cultured with recipient cells for 24 h before the RFP positive recipient cells were gated and analyzed by flow cytometry.
