# Supplementary material for: Involvement of Tetraspanin C189 in Cell-to-Cell Spreading of the Dengue Virus in C6/36 Cells
Source: PLoS Negl Trop Dis. 2015 Jul 1;9(7):e0003885. doi: 10.1371/journal.pntd.0003885 (PMC4488468; doi:10.1371/journal.pntd.0003885)
Supplement: S1 Table — (DOCX) [file pntd.0003885.s008.docx]

**S1 Table. The list of primer pairs used for constructs in the related experiments.**

|  |  |  | |
| --- | --- | --- | --- |
| **Primer** | **Orientation** | **Sequences, 5'-3'** |  |
| **189exF-SalI** | forward | AGGGGTCGACCATGGCACTGAATTGTGGATTATC |  |
| **189exR-NotI** | reverse | ATAGTTTAGCGGCCGCTCAAGCTTAAGCGTATCTTCTC |  |
| **189F2** | forward | GCGCATCGAGAGGGAAAG |  |
| **189R5** | reverse | GTGGAATCAACGCATACCAATG |  |
| **anti-189F** | forward | AAAGATATCATGGCACTGAATTGTGGAC |  |
| **anti-189R** | reverse | AAAGGATCCTCAAGCGTCAACATCAAGC |  |
| **189-F-KpnI** | forward | ATAGGTACCATGGCACTGAATTGTGGATTATC |  |
| **189-R-KpnI** | reverse | TATGGTACCTTAGCGTATCTTCTCTGTTGATTG |  |
| **C189-243 F** | forward | CTGCATGACCACGACCTATGG |  |
| **C189-311 R** | reverse | AGAGCGGCAACGACGATTT |  |
| **EcoRV-HAC189-F** | forward | AAAGATATCAATGGCACTGAATTGTGGATTATC |  |
| **HAC189a-XhoI-R** | reverse | TTTCTCGAGTTAAGCGTATCTTCTCTGTTGATTG |  |
| **miC189-83 bottom** | forward | CCTGAAACGCCTCCGTACCACCAGTCAGTCAGTGGCCAAAACTGGTGGTAATCGGAGGCGTTTC |  |
| **miC189-83 top** | reverse | TGCTGAAACGCCTCCGATTACCACCAGTTTTGGCCACTGACTGACTGGTGGTACGGAGGCGTTT |  |
| **EcoRI-mi-F** | forward | AAAGAATTCCTAGTTAAGCTATCAACAAGTTTG |  |
| **mi-R-Not I** | reverse | TTTGCGGCCGCATCAACCACTTTGTACAAGAAAG |  |
| **eGFP-F-KpnI** | forward | CGGGGTACCATGGTGAGCAAGGGCGAGG |  |
| **eGFP-R-NotI** | reverse | TTTGCGGCCGCTTACTTGTACAGCTCGTCC |  |
| **XhoI-eGFP-F** | forward | AAACTCGAGATGGTGAGCAAGGGCGAGGA |  |
| **eGFP-ApaI-R** | reverse | AAAGGGCCCTTACTTGTACAGCTCGTCCATG |  |
| **EcoRV-HAeGFP-F** | forward | AAAGATATCAATGGTGAGCAAGGGCGAGG |  |
| **HAeGFP-XhoI-R** | reverse | TTTCTCGAGTTACTTGTACAGCTCGTCCATG |  |
| **EcoRI-RFP-F** | forward | AAAGAATTCATGGTGTCTAAGGGCGAAGAG |  |
| **RFP-NotI-R** | reverse | TTTGCGGCCGCTTAATTAAGTTTGTGCCCCAGTTTG |  |
| **Q18SF** | forward | AGGTCCGTGATGCCCTTAGA |  |
| **Q18SR** | reverse | TACAATGTGCGCAGCAACG |  |
| **18SF** | forward | TGACTCAACACGGGAAAAC |  |
| **18SR** | reverse | CAGAACATCTAAGGGCATCAC |  |
| **KpnI-HA-EcoRI-F** | forward | CATGTACCCATACGATGTTCCAGATTACGCTCG |  |
| **KpnI-HA-EcoRI-R** | reverse | AATTCGAGCGTAATCTGGAACATCGTATGGGTACATGGTAC |  |
| **GRP94-EcoRV-F** | forward | AAAGATATCATGAAGTACCTGCTGCTTCTG |  |
| **GRP94-∆HDEL-HA-NotI-R** | reverse | AAAGCGGCCGCAAAGCGTAGTCTGGGACGTCGTATGGGTACTCGGCATCATCGTCGG |  |
| **Bip-EcoRV-F** | forward | AAAGATATCATGAAGCTGCTAGTACCGTTGGCCC |  |
| **Bip-∆KDEL-HA-NotI-R** | reverse | AAAGCGGCCGCAAAGCGTAGTCTGGGACGTCGTATGGGTAGAGATCGTCATCTTCGCCGGCAG |  |
